# Hibiscus Leachate Dye-Based Low-Cost and Flexible Dye-Sensitized Solar Cell Prepared by Screen Printing

**DOI:** 10.3390/ma14112748

**Published:** 2021-05-22

**Authors:** Congjun Cao, Tianning Yang, Guangxue Chen

**Affiliations:** 1 School of Printing, Packaging Engineering and Digital Media Technology, Xi’an University of Technology, Xi’an 710048, China; caocongjun@xaut.edu.cn (C.C.); tn_0628@163.com (T.Y.); 2 State Key Laboratory of Pulp and Paper Engineering, School of Light Industry and Engineering, South China University of Technology, Guangzhou 510640, China

**Keywords:** dye-sensitized solar cell, low-cost, hibiscus, screen printing

## Abstract

Although the price of dye-sensitized solar cells is lower than other solar cells, they still contain some high-cost materials, such as transparent conductive substrates, dyes (ruthenium dyes, organic dyes, etc.), and platinum counter electrodes. To solve this problem, a dye-sensitized solar cell based on hibiscus leaching solution and carbon black–silver electrodes was prepared by screen printing. The prepared low-cost dye-sensitized solar cells were flexible. The open-circuit voltage (V_oc_) of the obtained dye-sensitized solar cell is 0.65 V, the current density (J_sc_) is 90 μA/cm², and the fill factor (FF) is 0.241.

## 1. Introduction

Recently, silicon-based solar cells, the most efficient type of solar cell, have been widely used and industrially produced. However, the high cost and cumbersome preparation process of pure silicon have severely restricted its practical application for civilian applications. Therefore, it is of great significance to explore new efficient and low-cost solar cells [1,2,3]. One of the outstanding advantages of dye-sensitized solar cells is their low price [4]. Among the common dye-sensitized cells, the most expensive materials are dyes (ruthenium dyes (N3, N719, etc.) [5,6], organic dyes (carbazole dyes and oligothiophene dyes) [7]), transparent conductive substrates (ITO (indium tin oxide), FTO (SnO_2_:F), etc.), and the platinum-plated counter electrode. Recently, increasing attention has been paid to the plant dye hibiscus leach solution and carbon black–silver electrodes, with its advantages of being low-cost [8]. Although there is a gap in the current between the dye-sensitized solar cell and common dye-sensitized solar cells, the cost is only a few tenths of them. The voltage is almost the same, and there are three main reasons for the lower performance: the first is that the dye has not been purified to reduce costs, the second is that the performance of the hibiscus leachate dye itself is relatively poor, and the third is that the addition of a thickener causes the performance of the solar cell to decrease. Screen printing is highly suitable for large-scale production [9,10,11]. To make the layer printable, a diverse range of flexible electronic devices on a transparent substrate has recently been investigated. Flexibility is now a major trend in the development of printed electronics [12,13], and the substrates used in this article are flexible materials, meaning that the obtained dye-sensitized solar cells are also flexible.

**Figure 1:**
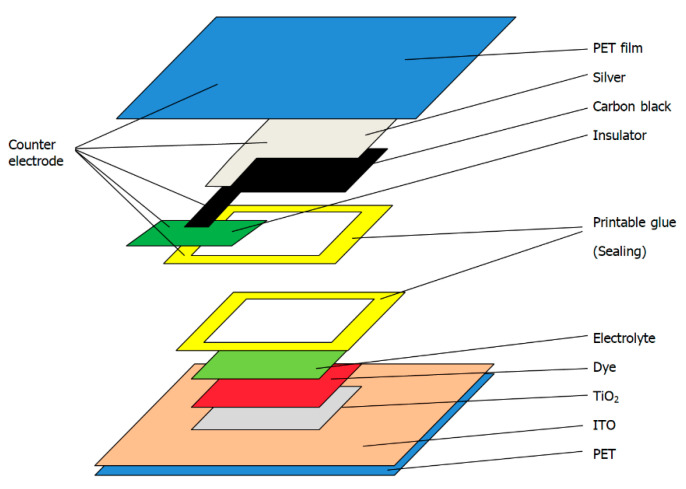
Structure of the flexible dye-sensitized solar cell.

The structure of the dye-sensitized solar cell is shown in Figure 1. The dye-sensitized solar cell consists of a transparent conductive substrate, TiO_2_, an electrolyte, dye, and electrodes. Most of the transparent conductive substrates are glass plated with a layer of ITO or FTO. Some people may also use PEDOT: PSS as a transparent conductive material [14,15,16], but its performance is inferior to ITO or FTO. Since ITO-plated PET film is flexible and more suitable for printing than ITO glass, ITO-plated PET film was selected.

In this paper, two types of TiO_2_ are used: one is the special titanium dioxide photocatalyst paste Kronoclean 7404, and the other is P25 TiO_2_ powder. The ideal dye should have a large spectral absorption range and a high molar extinction coefficient and be rich in oxygen-containing active functional groups for better attachment to the semiconductor porous layer. It should also have an energy level that matches the conduction band of the semiconductor to achieve electron injection. Furthermore, it should have good photochemical stability [17,18,19,20]. Hibiscus is a common plant, which is often used to soak in water to drink. It also functions as a dye, and its cost is less than 5% compared with expensive dyes. In order to prevent the dye from reacting with water, the electrolyte used in common dye-sensitized solar cells is mostly an organic solvent; that is, the redox couple (I^−^/I_3_^−^) is dissolved in an organic solvent such as acetonitrile [21]. Hibiscus is a natural plant, so there is no need to consider the type of solvent.

As is commonly known, the platinum-plated electrode has the best performance, good stability, and a certain catalytic effect [22]. However, the disadvantage is the high cost. As the metal with the best electrical conductivity, the price of silver is only 3% of that of platinum. Therefore, a carbon black–silver counter electrode was used, consisting of a layer of silver oriented on the bottom and then a layer of carbon black printed on the surface, which helps to protect the silver electrode from being corroded by the electrolyte and increases the durability and stability of the counter electrode [23].

The common sealing material used for glass substrates of dye-sensitized solar cells is the expensive sarin film. The adaptability of flexible substrates to glue is much better than that of glass, and the printable hot melt glue could be used as the sealing material. The sealing layer is printed after the other layers are printed. The glue is more uniform via printing than manual coating, and the amount is also easy to control.

## 2. Materials and Methods

### 2.1. Materials

The following materials were used: potassium iodide (KI, >99.5%, Carl Roth Gmbh & Co. KG, Karlsruhe, Germany); sodium chloride (NaCl, >99.5%, Carl Roth Gmbh & Co. KG, Karlsruhe, Germany); iodine (I_2_, >99.5%, Carl Roth Gmbh & Co. KG, Karlsruhe, Germany); acetonitrile (ACN, AR, Carl Roth Gmbh & Co. KG, Karlsruhe, Germany); carboxymethylcellulose sodium (NaCMC, >99.5%, Carl Roth Gmbh & Co. KG, Karlsruhe, Germany); polyvinyl pyrrolidone (PVP, >99.5%, Carl Roth Gmbh & Co. KG, Karlsruhe, Germany); polyethylene glycol 600 (PEG600, >99.5%, Carl Roth Gmbh & Co. KG, Karlsruhe, Germany); terpineol (AR, Carl Roth Gmbh & Co. KG, Karlsruhe, Germany); ethanol (AR, Carl Roth Gmbh & Co. KG, Karlsruhe, Germany); butanediol (AR, Carl Roth Gmbh & Co. KG, Karlsruhe, Germany); P25 TiO_2_ (>99.9%, Degussa Sonne/Mond Goldhandel GmbH & Co. KG, Berlin, Germany); Kronoclean 7404 (KRONOS Worldwide Inc., Dallas, TX, USA); Encor 1230s (ARKEMA Innovative Chemistry Co., Ltd., Paris, France); hibiscus (Topfruits Naturprodukte Co., Ltd., Ubstadt-Weiher, Germany); Simulgel (Seppic SA Co., Ltd., Paris, France); Stellmittel (Marabu GmbH & Co. KG, Tamm, Germany); xanthan gum (Spinnrad GmbH & Co. KG, Berlin, Germany); Borchi gel (OMG Borchers GMBH & Co. KG, Langenfeld, Germany); Luvogel (Lehmann & Voss & Co. KG, Hamburg, Germany); UV light curing ink: UV insolation green: BA51 (Sun Chemical Corporation Co., Ltd., Parsippany, NJ USA); hot melt glue: Kiwotherm L110 (Kissel + Wolf GMBH & Co. KG, Wiesloch, Germany); and silver paste and carbon paste (Hochschule der Medien Stuttgart and Varta GMBH & Co. KG, Hagen, Germany, respectively). Other materials were prepared in the laboratory.

### 2.2. Preparation Process

#### 2.2.1. Pretreatment of Transparent Conductive Substrate

The substrate cannot be heated with high temperature; titanium dioxide can only be sintered at low temperature, which will inevitably lead to a decrease in performance [24]. The substrate was ultrasonically cleaned with ethanol, then dried with nitrogen, and heated at 125 °C for 30 min. This is because the binder in the TiO_2_ paste needs to be heated to 120 °C for consolidation. Preheating the substrate at high temperature can prevent shrinkage of the substrate. The ITO surface of the substrate was then treated with plasma to increase the surface energy, which can increase its adsorption capacity for the paste.

After the various treatments, the square resistance of the substrate was measured, which should be between 30–40 Ω/□; substrates above this range were discarded to control variables.

#### 2.2.2. Preparation of TiO_2_ Layer

In order to enhance the water resistance of the Kronoclean 7404 and prevent the titanium dioxide layer from being damaged after the printing of dye and electrolyte, it was necessary to add a binder to the paste. The binder Encor 1230s was selected. Since both the TiO_2_ and the binder were liquid, there was no need to add a solvent. The mass fraction of the Encor 1230s was 30 wt.%. The formula of the prepared P25 TiO_2_ paste was 2.0 g P25 TiO_2_ powder, 8.9 g terpineol, 0.4 g PEG600 and 0.6 g ethyl cellulose.

A small amount of butylene glycol (less than 5 wt.%) was added into the slurry to slow down the drying speed and make the screen easy to clean after printing.

The TiO_2_ layer was printed first, and the thickness of the printed layer was about 10–15 μm. After printing, it was heated at 120 °C for 30 min for the bindings of the TiO_2_.

#### 2.2.3. Preparation of Dye Layer

In this paper, the dyes were hibiscus leachate with water and hibiscus leachate with ethanol. The dye layer was printed on the top of the TiO_2_. This layer then needed to be printed three times after drying. The color was light due to the amount of the dye being insufficient for one time of printing. In fact, the color did not get significantly darker even after three or more times printing.

For the printing of the counter electrode, a PET film with a thickness of 100 μm was used as the substrate. The size of the PET film was the same as that of the transparent conductive substrate. Similarly, since the carbon black layer and the silver layer needed to be heated, the PET film also needed to be preheated. However, the heat resistance of PET film is relatively poor, and so if the temperature is too high, it will cause the carbon black and silver to shrink and deform the electrode. The preheating temperature was therefore chosen as 110 °C. After 30 min preheating and cooling, the silver was printed, and then it was heated at 105 °C for 30 min. The carbon black was printed after cooling and then heated at 100 °C for 30 min. Next, an insulating layer needed to be printed to prevent the counter electrode from contacting the ITO and causing a short circuit. The insulating layer was printed with UV light curing ink and completed by a UV light curing machine after printing.

#### 2.2.4. Preparation of Electrolyte Layer

The electrolyte layer was prepared by screen printing. After printing a layer of electrolyte on the dye layer, a separator with a non-woven material of the same size as the TiO_2_ layer was added, and then a second layer of electrolyte was printed on the separator. The purpose of this process was to increase the amount of electrolyte in the dye-sensitized solar cell in order to avoid the problem of poor contact of the photoanode and the counter electrode. This process also serves to mitigate electrolyte volatilization, thereby reducing the degradation of battery performance. Because the electrolyte needed to be kept wet, the thickness of the electrolyte layer could not be increased if the second layer was printed directly. After adding a separation layer, the thickness of the electrolyte remained unchanged during printing after drying. This greatly improved the performance of the solar cells.

#### 2.2.5. Preparation of the Dye-Sensitized Solar Cells

First, the sealing layer was printed on both the transparent conductive substrate and the counter electrode. This was left to dry naturally.

**Figure 2:**
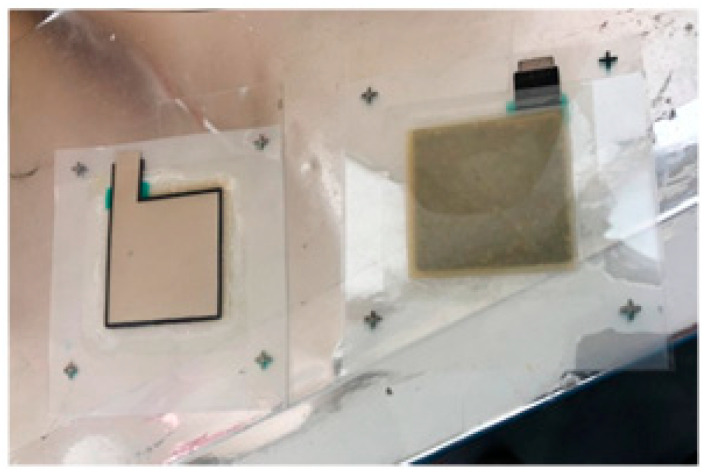
Manufactured dye-sensitized solar cells.

The transparent conductive substrate was then printed with each functional layer and the counter electrode joined together. The sealing part was heated to melt and bond, as shown in Figure 2. The prepared dye-sensitized solar cells were then complete.

## 3. Characterizations

Scanning electron microscopy (SEM) was achieved using a field emission scanning electron microscope (SU-8000, Hitachi Co., Ltd., Tokyo, Japan). SEM images characterized the surface of the material and indirectly reflected the quality (flatness and continuity) of printing, the magnification of which was 8000 and 50,000 times, respectively. The accelerating voltage was 3 kV, and the samples were sputter-coated with gold prior to the test.

The JV curve (one of the most important performance indicators of solar cells) was measured by Electrochemical Workstation (BioLogic BCS-810, BioLogic Sciences Instruments, Seyssinet-Pariset, France).

The square resistance of the transparent conductive substrate and the counter electrode were measured with a four-point resistance meter (FT331, Rooko Instrument Co., Ltd. Ningbo, China).

The conductivity of the electrolyte was measured with a conductivity meter (Konduckometer 702, Knick Elektronische Messgeräte GmbH & Co. KG, Berlin, Germany).

The absorbance and transmittance of ultraviolet and visible light were measured by UV–Vis spectrophotometer (UV1800, Shimadzu Corporation, Kyoto, Japan).

## 4. Results

Since this paper is the realization of the full printing of a dye-sensitized solar cell, the results and discussion are also analyzed from the structure of each layer.

### 4.1. TiO_2_ Layer

**Figure 3:**
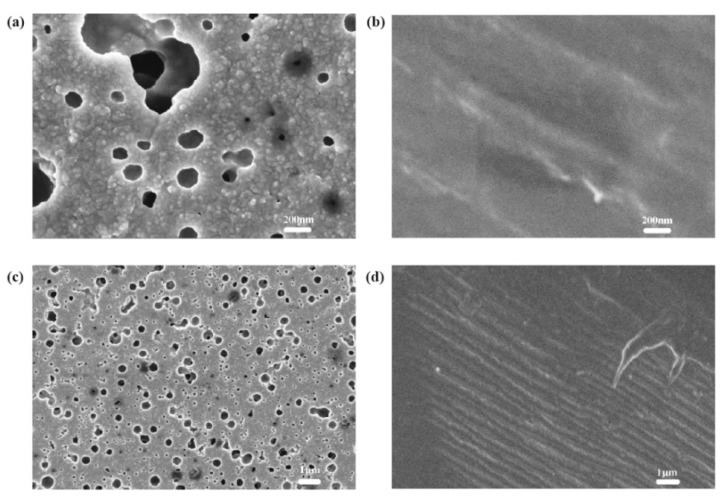
SEM images of the TiO_2_ layer prepared by Kronoclean 7404: (**a**,**c**) are the surface with a magnification of 50,000 times and 8000 times, respectively; (**b**,**d**) are the cross-section with a magnification of 50,000 times and 8000 times, respectively.

**Figure 4:**
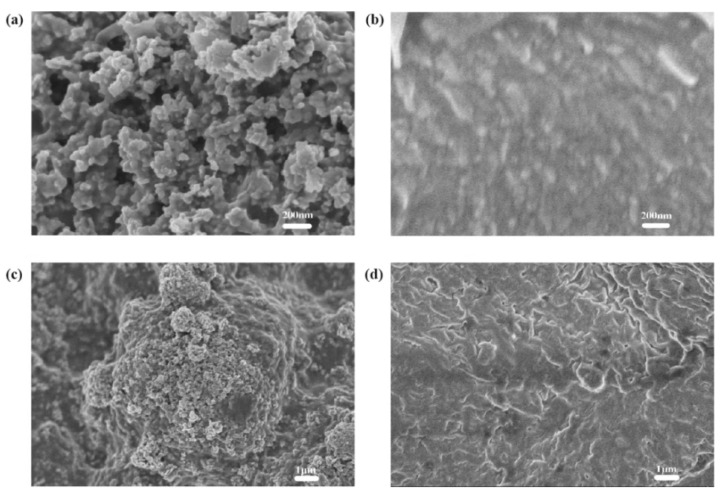
SEM images of the TiO_2_ layer prepared by P25 TiO_2_ powder: (**a**,**c**) are the surface with a magnification of 50,000 times and 8000 times, respectively; (**b**,**d**) are the cross-section with a magnification of 50,000 times and 8000 times, respectively.

The V_oc_ and J_sc_ of the dye-sensitized solar cells with Kronoclean 7404 paste are higher than dye-sensitized solar cells with P25 TiO_2_ paste. As shown in Figure 3 and Figure 4 (the SEM images of the TiO_2_ layer) the TiO_2_ layer prepared by Kronoclean 7404 paste has more holes after low-temperature sintering, suggesting that it could absorb more dyes and perform better. Dyes are the heart of dye-sensitized solar cells, and their quantity and performance directly affect the performance of solar cells. Therefore, the performance of dye-sensitized solar cells with Kronoclean 7404 paste is better.

### 4.2. Dye Layer

**Figure 5:**
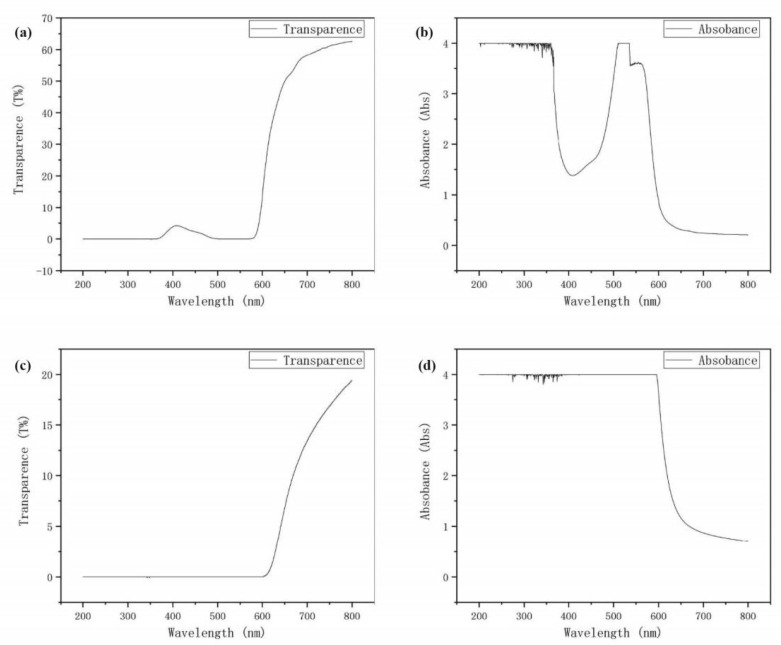
Ultraviolet-visible light transmittance (**a**) and absorbance (**b**) of the leachate with ethanol of dried hibiscus flower and the visible light transmittance (**c**) and absorbance (**d**) of the leachate with water.

The absorbance and transmittance of the dyes are important parameters to characterize its performance. Since natural dyes do not require organic solvents, water and ethanol are used to extract the hibiscus separately. It can be seen from Figure 5 that when the wavelengths are 0–350 nm and 500–550 nm, the transmittance and absorbance of the leachate with water and the leachate with ethanol are similar, while at other wavelengths, the transmittance of the leachate with ethanol is higher than that of the leachate with water. The absorbance is lower than that of the leachate with water. This proves that the hibiscus leachate with water can absorb more sunlight. It is also confirmed by subsequent experiments (JV curve) that the performance of the leachate with water is better than that of the leachate with ethanol.

**Figure 6:**
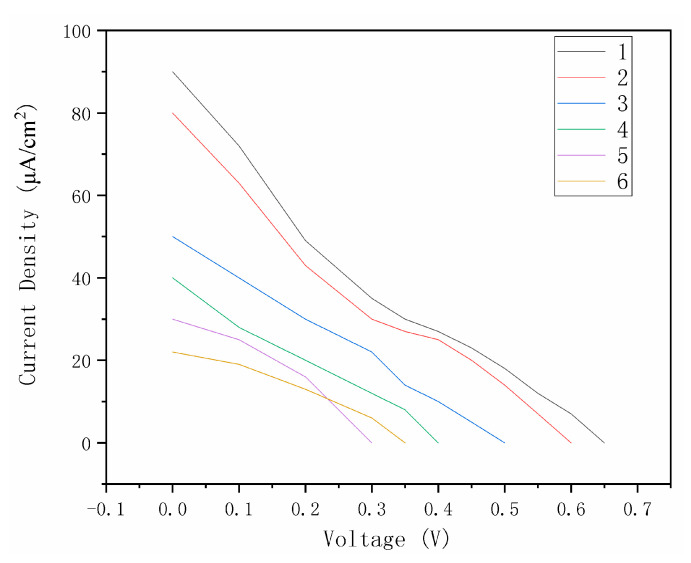
Performance of different thickeners in the dye layer (1:7 wt.% Simulgel with water; 2:8 wt.% xanthan gum with water; 3:12 wt.% ethyl cellulose with ethanol; 4:5 wt.% Stellmittel with ethanol; 5:5 wt.% Stellmittel with water; 6:20 wt.% Borchi gel with ethanol).

It is undeniable that after adding any kind of thickener, the performance of the obtained dye-sensitized solar cell decreases. As per Figure 6, the JV curves of different thickeners in dye layer indicate that the performance of Simulgel with 7 wt.% is the best (the thickener of the electrolyte is 5 wt.% xanthan gum). V_oc_, Jsc, and FF for Figure 6 are in Table 1.

### 4.3. Electrolyte Layer

As shown in Table 2, the conductivity of the NaCl aqueous solution is the highest in the electrolyte when the mass fraction is the same. Furthermore, the amount of NaCl dissolved in water is much lower than that of KI. The conductivity of the KI aqueous solution is nearly twice that of NaCl aqueous solution. The performance of the prepared dye-sensitized solar cell is very poor due to the poor conductivity of the electrolyte in acetonitrile.

Therefore, saturated KI aqueous solution is used as an electrolyte, and 0.1 mol/L I_2_ is added to form a redox couple (I^−^/I_3_^−^).

**Figure 7:**
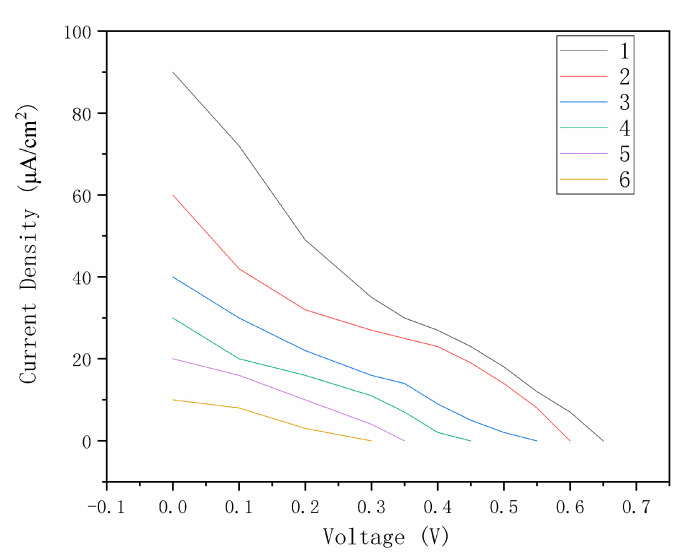
Performance of different thickeners in electrolyte layer (1:6 wt.% xanthan gum with water; 2:5 wt.% Simulgel with water; 3:6 wt.% NaCMC with water; 4:3 wt.% Stellmittel with water; 5:20 wt.% PVP10000 with water; 6:25 wt.% Luvogel with water).

The electrolyte also needs a thickener added to make it printable. Due to the poor performance of acetonitrile solvent, the water solvent thickener is considered. Figure 7 shows the performance of different thickeners in the electrolyte layer, where the best of the different thickeners is 5 wt.% xanthan gum (the thickener of the dye is about 7 wt.% Simulgel). V_oc_, Jsc, and FF for Figure 7 are in Table 3.

### 4.4. Counter Electrode

**Figure 8:**
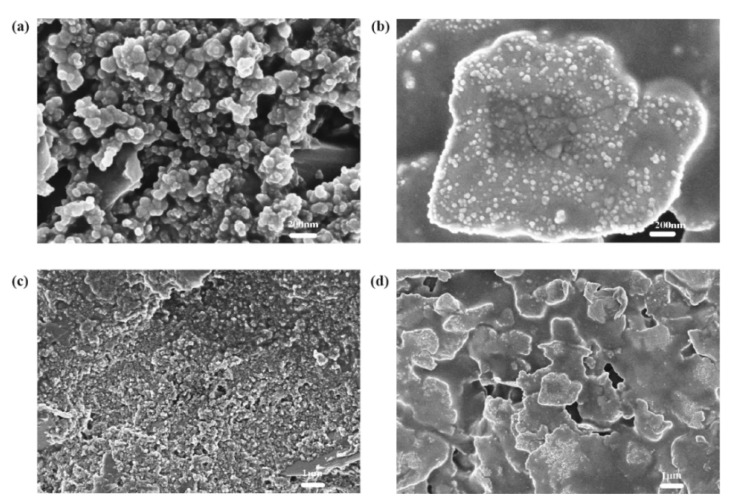
SEM images of carbon black layer and the silver layer: (**a**,**c**) are the surface of carbon black layer with a magnification of 50,000 times and 8000 times, respectively; (**b**,**d**) are the surface of silver layer with a magnification of 50,000 times and 8000 times, respectively.

From the SEM images of the counter electrode (Figure 8), it can be found that the carbon particles and the scaly silver particles of the counter electrode are well connected. Using the four-point resistance meter for measurement, the square resistance was about 10–12 Ω/□. Therefore, the carbon black–silver counter electrode prepared by screen printing has good performance. 

## 5. Conclusions

In conclusion, we report a low-cost, fully printed, and flexible dye-sensitized solar cell. The dye-sensitized solar cell consists of transparent conductive substrate (ITO) plated PET film, a Kronoclean 7404 TiO_2_ layer (Kronoclean 7404), the dye (hibiscus leachate with water), the electrolyte (saturated KI aqueous solution with 0.1 mol/L I_2_), and the counter electrode (carbon black–silver electrode). The maximum V_oc_ of the dye-sensitized solar cell obtained is 0.65 V, the I_sc_ is 90 μA/cm^2^ and the FF is 0.241. The prepared dye-sensitized solar cells are not only low-cost but also use a straightforward screen-printing process suitable for industrial production. We believe that our methodology encourages the development of a series of dye-sensitized solar cells that can be widely used for future flexible devices.

## Figures and Tables

**Table 1:** V_oc_, Jsc, and FF for Figure 6.

	1	2	3	4	5	6
V_oc_	0.65 V	0.60 V	0.50 V	0.40 V	0.30 V	0.35 V
J_sc_	90 μA/cm^2^	80 μA/cm	50 μA/cm	40 μA/cm	30 μA/cm	22 μA/cm
FF	0.241	0.239	0.256	0.262	0.413	0.344

**Table 2:** Conductivity of the electrolyte in NaCl or KI solution.

Concentration		Conductivity (S)	
	NaCl (Water)	KI (Water)	KI (Acetonitrile)
5%	79	51	/
10%	138	99	/
15%	191.5	143	/
20%	225	185	/
30%	/	268	/
40%	/	314	/
50%	/	404	/
Saturated	251	482	12.6

**Table 3:** V_oc_, Jsc, and FF for Figure 7.

	1	2	3	4	5	6
V_oc_	0.65 V	0.60 V	0.55 V	0.45 V	0.35 V	0.30 V
J_sc_	90 μA/cm^2^	60 μA/cm	40 μA/cm	30 μA/cm	20 μA/cm	10 μA/cm
FF	0.241	0.283	0.247	0.265	0.302	0.276

## Data Availability

Data is contained within the article and can be requested from the corresponding author.

## References

[1] Yoshikawa K., Kawasaki H., Yoshida W., Irie T., Konishi K., Nakano K., Uto T., Adachi D., Kanematsu M., Uzu H. (2017). Silicon heterojunction solar cell with interdigitated back contacts for a photoconversion efficiency over 26%. Nat. Energy.

[2] Jean J., Brown P.R., Jaffe R.L., Buonassisi T., Bulovic V. (2015). Pathways for solar photovoltaics. Energy Environ. Sci..

[3] Schultz O., Glunz S.W., Willeke G.P. (2012). Multicrystalline silicon solar cells exceeding 20% efficiency. Prog. Photovolt. Res. Appl..

[4] O’Regan B., Graetzel M. (2010). A low-cost, high-efficiency solar cell based on dye-sensitized colloidal TiO_2_ films. Nat. Mater..

[5] Nazeeruddin M.K., Pechy P., Graetzel M. (1997). Efficient panchromatic sensitization of nanocrystalline TiO_2_ films by a black dye based on a trithiocyanato-tuthenium complex. Chem. Commun..

[6] Antonio A., Planells M., Hollman D.J., Stranks S.D., Petrozza A., Kandada A.R.S., Vaynzof Y., Pathak S.K., Robertson N., Snaith H.J. (2014). An organic “donor-free” dye with enhanced open-circuit voltage in solid-state sensitized solar cells. Adv. Energy Mater..

[7] Omar A., Fakir M.S., Hamdan K.S., Hamdan K.S., Rased N.H., Rahim N.A. (2020). Chemical, optical and photovoltaic properties of TiO_2_/reduced graphene oxide photoanodes sensitized with Roselle and N719 dyes for dye-sensitized solar cell application. Pigment. Resin Technol..

[8] Murakami T.N., Kay A., Ito S., Wang Q. (2006). Highly efficient dye-sensitized solar cells based on carbon black counter electrodes. J. Electrochem. Soc..

[9] Wen Y., Xu J. (2017). Scientific Importance of water-processable PEDOT:PSS and preparation, challenge and new application in sensors of its film electrode: A Review. J. Polym. Sci. Part. A Polym. Chem..

[10] Lorenz A., Senne A., Rohde J., Kroh S., Wittenberg M., Krueger K., Clement F., Biro D. (2015). Evaluation of flexographic printing technology for multi-busbar solar cells. Energy Procedia.

[11] Krebs F.C. (2009). Polymer solar cell modules prepared using roll-to-roll methods: Knife-over-edge coating, slot-die coating and screen printing. Sol. Energy Mater. Sol. Cells.

[12] Tran T.S., Dutta N.K., Choudhury N.R. (2018). Graphene inks for printed flexible electronics: Graphene dispersions, ink formulations, printing techniques and applications. Adv. Colloid Interface Sci..

[13] Kim S., Moon H., Kwon H., Lee G., Yu D., Choi J., Park J., Kim S.J., Yoo S. (2019). Organic Vapor-Jet Printing with Reduced Heat Transfer for Fabrication of Flexible Organic Devices. Adv. Mater. Technol..

[14] Song D., Li M., Jiang Y., Chen Z., Bai F., Li Y., Jiang B. (2014). Facile fabrication of MoS2/PEDOT: PSS composites as low-cost and efficient counter electrodes for dye-sensitized solar cells. J. Photochem. Photobiol. A Chem..

[15] Ke C.R., Chang C.C., Ting J.M. (2015). Modified conducting polymer films having high catalytic activity for use as counter electrodes in rigid and flexible dye-sensitized solar cells. J. Power Sources.

[16] Wu K., Ma J., Cui W., Ruan B., Wu M. (2017). The impact of metal ion doping on the performance of flexible poly(3,4-ethylenedioxythiophene) (PEDOT) cathode in dye-sensitized solar cells. J. Photochem. Photobiol. A Chem..

[17] Ragoussi M.E., Torres T. (2015). New generation solar cells: Concepts, trends and perspectives. Chem. Commun..

[18] Sekar N., Gehlot V.Y. (2010). Metal complex dyes for dye-sensitized solar cells: Recent development. Resonance.

[19] Horiuchi T., Miura H., Sumioka K., Uchida S. (2004). High efficiency of dye-sensitized solar cells based on metal-free indoline dyes. J. Am. Chem. Soc..

[20] Bella F., Vlachopoulos N., Nonomura K., Zakeeruddin S.M., Grätzel M., Gerbaldi C., Hagfeldt A. (2015). Direct light-induced polymerization of cobalt-based redox shuttles: An ultrafast way towards stable dye-sensitized solar cells. Chem. Commun..

[21] Chen M., Shao L. (2016). Review on the recent progress of carbon counter electrodes for dye-sensitized solar cells. Chem. Eng. J..

[22] Ghifari A., Long D.X., Kim S., Ma B., Hong J. (2020). Transparent Platinum Counter Electrode Prepared by Polyol Reduction for Bifacial. Dye-Sensitized Sol. Cells Nanomater..

[23] Lee W.J., Ramasamy E., Lee D.Y., Song J.S. (2006). Glass frit overcoated silver grid lines for nano-crystalline dye sensitized solar cells. J. Photochem. Photobiol. A Chem..

[24] Liu J., Li Y., Arumugam S., Tudor J., Beeby S. (2018). Investigation of low temperature processed titanium dioxide (TiO_2_) films for printed dye sensitized solar cells (DSSCs) for large area flexible applications. Mater. Today Proc..

